# Examining the Feasibility of Implementing Digital Mental Health Innovations Into Hospitals to Support Youth in Suicide Crisis: Interview Study With Young People and Health Professionals

**DOI:** 10.2196/51398

**Published:** 2023-11-16

**Authors:** Demee Rheinberger, Rachel Baffsky, Lauren McGillivray, Isabel Zbukvic, Ann Dadich, Mark Erik Larsen, Ping-I Lin, Daniel Z Q Gan, Catherine Kaplun, Holly C Wilcox, Valsamma Eapen, Paul M Middleton, Michelle Torok

**Affiliations:** 1 Black Dog Institute University of New South Wales Sydney, NSW Australia; 2 Orygen Parkville, Victoria Australia; 3 Centre for Youth Mental Health The University of Melbourne Parkville, Victoria Australia; 4 School of Business Western Sydney University Parramatta, NSW Australia; 5 Centre for Big Data Research in Health University of New South Wales Sydney, NSW Australia; 6 Neuroscience Research Australia University of New South Wales Sydney, NSW Australia; 7 Mental Health Research Unit The Ingham Institute for Applied Medical Research Liverpool, NSW Australia; 8 Academic Unit of Infant Child and Adolescent Services (AUCS) South Western Sydney Local Health District Liverpool, NSW Australia; 9 Transforming Early Education and Child Health (TeEACH) Research Centre Western Sydney University Sydney, NSW Australia; 10 The Ingham Institute for Applied Medical Research Liverpool, NSW Australia; 11 Department of Mental Health Johns Hopkins Bloomberg School of Public Health Baltimore, MD United States; 12 South Western Sydney Clinical School University of New South Wales Sydney, NSW Australia; 13 Department of Emergency Medicine Liverpool Hospital Liverpool, NSW Australia; 14 South Western Emergency Research Institute (SWERI) Ingham Institute Liverpool, NSW Australia

**Keywords:** mobile health, mHealth, digital health, mental health, suicide prevention, self-harm, young people, hospitals, mobile phone

## Abstract

**Background:**

Hospitals are insufficiently resourced to appropriately support young people who present with suicidal crises. Digital mental health innovations have the potential to provide cost-effective models of care to address this service gap and improve care experiences for young people. However, little is currently known about whether digital innovations are feasible to integrate into complex hospital settings or how they should be introduced for sustainability.

**Objective:**

This qualitative study explored the potential benefits, barriers, and collective action required for integrating digital therapeutics for the management of suicidal distress in youth into routine hospital practice. Addressing these knowledge gaps is a critical first step in designing digital innovations and implementation strategies that enable uptake and integration.

**Methods:**

We conducted a series of semistructured interviews with young people who had presented to an Australian hospital for a suicide crisis in the previous 12 months and hospital staff who interacted with these young people. Participants were recruited from the community nationally via social media advertisements on the web. Interviews were conducted individually, and participants were reimbursed for their time. Using the Normalization Process Theory framework, we developed an interview guide to clarify the processes and conditions that influence whether and how an innovation becomes part of routine practice in complex health systems.

**Results:**

Analysis of 29 interviews (n=17, 59% young people and n=12, 41% hospital staff) yielded 4 themes that were mapped onto 3 Normalization Process Theory constructs related to coherence building, cognitive participation, and collective action. Overall, digital innovations were seen as a beneficial complement to but not a substitute for in-person clinical services. The timing of delivery was important, with the agreement that digital therapeutics could be provided to patients while they were waiting to be assessed or shortly before discharge. Staff training to increase digital literacy was considered key to implementation, but there were mixed views on the level of staff assistance needed to support young people in engaging with digital innovations. Improving access to technological devices and internet connectivity, increasing staff motivation to facilitate the use of the digital therapeutic, and allowing patients autonomy over the use of the digital therapeutic were identified as other factors critical to integration.

**Conclusions:**

Integrating digital innovations into current models of patient care for young people presenting to hospital in acute suicide crises is challenging because of several existing resource, logistical, and technical barriers. Scoping the appropriateness of new innovations with relevant key stakeholders as early as possible in the development process should be prioritized as the best opportunity to preemptively identify and address barriers to implementation.

## Introduction

### Background

Self-harm (intentional self-injury or self-poisoning) is a leading contributor to suicide and disability among young people worldwide [1]. There have been considerable increases in hospital presentations for intentional self-harm among young Australians aged 10 to 24 years over the past decade [2], and this upward trend has only been exacerbated by the onset of the COVID-19 pandemic [3,4]. Research shows that young people are more likely to present to hospital rather than community-based services in times of acute suicide distress [5]; however, the hospital environment can be a negative experience for many [6]. Long wait times for assessment and treatment, limited access to trained mental health clinicians, and stigmatizing interactions with medical staff can lead to negative care experiences in hospitals, exacerbating distress [6,7]. Individuals who have experienced effective care in hospitals are more likely to engage with care in future suicide crises [8]. Therefore, as part of efforts to prevent repeat suicidal behavior [9], assessing the feasibility of new models of care to improve the quality of care in hospitals is important.

Digital therapeutic innovations (treatment via smartphones, tablets, or computers) provide an opportunity to cost-effectively support people in suicide crises (suicidal thoughts, plans, or behaviors) presenting to hospital, whereas their technology-enabled delivery models could concurrently improve hospital workflow and resource distribution [10]. There is increasing evidence in clinical and nonclinical populations that digital therapeutics or mobile health interventions can reduce suicidal thoughts and behaviors. Recent meta-analyses and stand-alone trials have shown that digital therapeutics that specifically target suicidality can reduce the severity of suicidal ideation in young people and adults [11,12]. Digital therapeutics can also significantly reduce symptoms of depression, psychological distress, and self-harm [13]—important risk factors for self-harm and suicidal behavior [14].

### Digital Therapeutics Integration: Theoretical Underpinnings

A critical first step in understanding whether and how digital therapeutics can be integrated as part of routine mental health care provision in hospitals is to consider the perspectives of stakeholders—the young people presenting to hospital in a suicide crisis and the hospital staff who provide care [15]. Exploring staff perspectives *before* implementation can provide information about potential attitudinal and structural barriers to and facilitators of system change [16]. It can also offer insights into strategies to overcome these barriers [17]. Furthermore, involving staff in the early phases of intervention design and development can increase the likelihood of adherence to and adoption of an intervention [18] by addressing staff concerns and hesitancy regarding changes early [19]. Currently, little is known about whether digital therapeutics would be suitable for delivery in complex hospital settings, how stakeholders make sense of these resources, and the strategies to support integration and adoption of digital care. To optimize the implementation potential of digital interventions in these settings, it may help to consider the factors that enable or inhibit new interventions to be incorporated (“normalized”) into routine practice through the lens of an implementation framework such as the Normalization Process Theory (NPT) [20].

NPT assumes that 4 key constructs determine the implementation of new practices or innovations: “coherence building” (the extent to which the practice makes sense and is perceived to have benefits for users and providers), “cognitive participation” (engagement with the new practice), “collective action” (the work or activities needed to support implementation), and “reflexive monitoring” (reviewing how the practice affects users and providers and making modifications) [20]. The latter is not relevant to the preimplementation stage and will not be discussed in this paper. NPT has been applied in numerous health settings, typically once an intervention has been developed and tested to assess its feasibility during real-world implementation [21,22]. However, applying NPT at the point of implementation may be too late in the process to fully address implementation barriers and achieve routine integration. For example, one study that “digitalized” an existing face-to-face child and adolescent mental health service did not adequately consider the existing needs and capacity of the service before the digital adaptation, resulting in implementation failure and wasted resources [23]. Examining how end users and providers understand and would engage with digital therapeutics as part of their routine practice can inform the design and development of solutions that are fit for purpose, optimizing uptake and sustainability before implementation takes place. Although health professionals identify digital therapeutics as a potential avenue to increase young people’s access to care, particularly where services might be lacking [24], there has been no research focused on defining and addressing care (intervention) and implementation needs in Australian hospitals to support young people in suicide crises.

### This Study

Addressing the knowledge gaps surrounding the potential benefits, barriers, and collective action required for digital therapeutics is the critical first step in designing acceptable and feasible interventions and implementation strategies for integration. Accordingly, we interviewed young people who had previously presented to a hospital for suicidal crises and health professionals who worked with these young people using the NPT framework to address the following research questions:

1. What are their attitudes toward digital therapeutics and perceived barriers?
2. How should digital therapeutics be provided to young people in hospitals and what factors might support engagement?
3. To what extent are digital therapeutics compatible with workplace practices and what strategies might be needed to optimize compatibility and support delivery?

## Methods

This manuscript was prepared in accordance with the COREQ (Consolidated Criteria for Reporting Qualitative Research) standards (Tong et al [25]).

### Recruitment

This point-in-time qualitative study involved one-on-one interviews with a convenience sample of “young people” from across Australia who had a lived or living experience of presenting to hospital for a suicidal crisis and “health professionals” who interact with young people who present for suicidal crises.

Young people were eligible to participate if they were aged 16 to 24 years (inclusive), were Australian residents, had access to an internet-connected computer or smartphone (for the interview), had presented to an Australian hospital for a suicide crisis (ideation or self-harm with suicidal intent) in the previous 12 months, and were able to speak and understand English fluently. For participant safety, young people were ineligible to participate if they were flagged as having an imminent suicide risk according to the 3-item Patient Safety Screener [26], which assesses for current suicidal behavior. These young people were offered the option of being contacted by a clinical psychologist based at the Black Dog Institute, Sydney, New South Wales, and were provided with a list of crisis resources.

Health professionals were eligible to participate if they were currently employed (part time or full time) in an Australian hospital (public or private) in an administrative or clinical role that interacted with young people experiencing mental health issues, had access to an internet-connected computer or smartphone, and were aged ≥18 years. There were no exclusion criteria for health professionals.

Separate targeted advertisements for young people and health professionals were posted on the Black Dog Institute’s website and their official social media channels (Twitter, Facebook, and Instagram) and promoted via their monthly e-newsletters. Recruitment took place between May 2022 and November 2022.

### Study Procedure

The web-based recruitment advertisements included a URL linked to a Qualtrics portal (Qualtrics International Inc) where interested persons were invited to complete an opt-in consent form followed by an eligibility screening process. To ensure that young people aged 16 or 17 years understood what they were consenting to, they completed a Gillick competency assessment [27] comprising 5 questions. Participants who either failed the Gillick competency task or were deemed ineligible based on other study criteria were directed to a web page that displayed relevant contact details for free 24-hour crisis support services (eg, Lifeline). Any individual flagged as actively suicidal during screening was asked an additional question as to whether they would like to be contacted by the research team’s clinical psychologist (yes or no). If yes, they were asked to provide a name and telephone number and informed that they would be contacted within the next 48 business hours between 9 AM and 5 PM.

Eligible participants had to register their details to proceed with the interview, including their name, email address, mobile telephone number, and preferred contact method. The research staff then contacted participants to schedule an interview. Participants were sent a reminder SMS text message or email a day before their scheduled interview and asked to reply “yes” to confirm or urged to contact the research team to reschedule if needed.

Interviews were conducted via the secure web-based Zoom platform (Zoom Video Communications), and all interviews were audio recorded with permission from the participants. Interviews were conducted between May 2022 and October 2022, inclusive, with young people and between November 2022 and December 2022, inclusive, with health professionals. Participant well-being was monitored throughout and checked at interview completion. If any participant expressed distress or discomfort, they were offered to speak with a clinical psychologist. Initial contact with the psychologist was made within 48 business hours. All participants were sent contact details for free 24/7 national crisis support services.

The first 14% (4/29) of the interviews with young people were conducted by 3 members of the research team in dyads (DR, MT, and LM) to ensure that the interview questions adequately addressed the study aims. Once it was determined that the questions provided relevant data, the remaining interviews (25/29, 86%) were conducted by a single researcher (DR). Interviews were conducted until saturation of information was reached. The audio recordings were deidentified before being sent to an external agency for professional transcription.

### Interview Guide

The interview guide was informed by NPT [20]. NPT can clarify the processes and conditions that influence whether and how an intervention becomes part of routine practice. It has guided numerous implementations of new practices in health services [28]. The interview guide addressed 3 NPT factors to understand the implementation context: coherence, that is, how people make sense of a practice (“How do you feel about digital therapeutics being offered as a care option in hospital? Why do you feel that way?”); cognitive participation, that is, how people engage with and support new practices (“What do you think would help or hinder the introduction of digital therapeutics in hospitals/your hospital?”); and collective action, that is, how new practices become integrated into routine practice (“When do you think digital therapeutics should be offered to young people in suicide crisis?”). Only health professionals were asked questions in relation to what had worked previously in their experience when new practices were implemented in hospitals (collective action).

Digital therapeutics were defined during the interviews as treatment delivered through any digital device—like a smartphone app or web-based computer program that can help you self-manage and improve a mental health concern. The treatments might be based on a therapeutic model (like cognitive behavioral therapy), skills-based to help you manage stress, or just have one focus like mindfulness or safety planning.

### Ethical Considerations

The study procedures were approved by the University of New South Wales Human Research Ethics Committee (HC210973). All participants completed opt-in informed consent procedures.

All audio-recorded files were pseudonymized and stored securely on a password-protected OneDrive (Microsoft Corp). Only those investigators named on the approved ethics application had password access to the audio files and transcripts. All data are reported anonymously.

All participants were compensated with an Aus $50 (US $31.53) e–gift voucher for their time, which was emailed to them following completion of the interview.

### Data Analysis

Framework analysis was used to construct findings deductively guided by NPT factors and inductively from the interviews [29,30]. Interview transcripts were then analyzed using the NVivo software (version 20; QSR International). The analysis commenced with 2 authors (DR and RB) familiarizing themselves with the interviews through extensive reading of the transcripts. Codes were developed by both authors independently through a line-by-line investigation of the transcripts. After 10 transcripts were coded (5 from each participant group), an analytical framework was developed by mapping the codes to the NPT factors (coherence, cognitive participation, and collective action). This framework was then applied to the remaining transcripts, and the codes and themes were refined through interrogation and discussions among the research team. This resulted in 4 themes separated into the 3 NPT factors assessed in the interviews. The themes and their codes were synthesized and reported alongside data extracts that represented the themes.

## Results

### Participant Sample

A total of 29 individuals participated in this study, comprising 17 (59%) young people and 12 (41%) health professionals.

#### Young People

The mean age of the young people was 18.4 (SD 2.9; range 16-24) years. Most (11/17, 65%) were female, followed by non-binary (4/17, 24%) and male (2/17, 12%). Most attended hospitals in New South Wales (14/17, 82%), followed by Western Australia (2/17, 12%) and Tasmania (1/17, 6%). Most attended hospitals located in metropolitan areas (11/17, 65%), with 18% (3/17) attending hospitals in regional areas and 18% (3/17) attending hospitals in rural areas. Of the 17 young people who participated, 13 (76%) had used a digital therapeutic at some point before the interview, and all but 1 (94%) identified that a digital therapeutic would be beneficial in a hospital.

#### Health Professionals

The mean age was 35.4 (SD 11.6; range 21-56) years. Health professionals worked in Victoria (5/12, 42%), New South Wales (3/12, 25%), Western Australia (3/12, 25%), and Queensland (1/12, 8%), with most working in regional areas (8/12, 67%) and 33% (4/12) working in metropolitan areas. All the health professionals worked in public hospitals or community services. Most health professionals were female (10/12, 83%), and only 17% (2/12) of the participants were male. The largest proportion of health professionals worked as nurses (5/12, 42%), followed by mental health clinicians (3/12, 25%), peer support workers (2/12, 17%), a hospital physician (1/12, 8%), and a mental health support worker (1/12, 8%). In total, 42% (5/12) engaged with suicidal adolescents exclusively in emergency departments, 25% (3/12) worked with young people in the community, 8% (1/12) worked with young people in general hospital wards, 8% (1/12) worked in both the emergency department and hospital wards, 8% (1/12) worked in hospital wards and the community, and 8% (1/12) worked in the emergency department and the community. More than half (7/12, 58%) had no experience recommending or using digital therapeutics in their practice.

### Thematic Analysis

#### Overview

In total, 4 themes (involving 15 individual codes) were mapped to 3 NPT constructs related to implementation mechanisms: shared sense making of digital therapeutics (“coherence building”), perceived barriers to digital therapeutic integration (“cognitive participation”), and compatibility considerations and strategies to enhance implementation (“collective action”; Table 1). A total of 64% (7/11) of the codes in the first 3 themes (Table 1) were articulated by both participant groups. Health professionals exclusively outlined implementation strategies (fourth theme).

**Table 1:** The 4 themes and related codes and the sample in which each code was identified, mapped to the Normalization Process Theory (NPT).

NPT factor, theme, and code			Reported by young person	Reported by health professional
**Coherence building**				
	**Shared sense making of the intervention’s purpose and its benefits beyond treatment as usual**			
		Complement to face-to-face care	✓	✓
		Digital therapeutic might be inappropriate in a crisis	✓	✓
		Digital therapeutic is always accessible	✓	
**Cognitive participation**				
	**Barriers to investing time, energy, and work on the intervention**			
		Limited access to information and communications technology	✓	✓
		Time and human resource constraints		✓
**Collective action**				
	**Compatibility considerations for integration into existing workplace practices or infrastructure**			
		Timing	✓	✓
		Supported use of digital therapeutics	✓	✓
		Framed as optional	✓	
	**What is needed for staff to integrate into practice**			
		Increasing staff support		✓
		Conducting ongoing training		✓
		Local opinion leaders		✓
		Informing staff of changes		✓

#### Theme 1: Shared Sense Making of the Intervention’s Purpose and Its Benefits Beyond Treatment as Usual (Coherence Building)

This theme clarified how health professionals and young people made sense of digital therapeutics in hospitals and how they envisioned digital therapeutic benefits being used during and in the aftermath of a suicide crisis in hospitals. The participants indicated how a digital therapeutic can support face-to-face care, concerns over the appropriateness of a digital therapeutic when a young person experiences a crisis, the accessibility of digital therapeutics, and the ease young people have using digital devices.

##### Complement to Face-to-Face Care

Both young people and health professionals commented that a digital therapeutic would be effective as an additional (adjunct) support for young people but should not replace face-to-face care in hospitals:

I think as a supplementary care they are great for before, during and after but as long as you get primary care.

Young person 05; aged 22 years

I obviously think it’s not the same kind of thing, like the benefits you get from both spaces. I don’t think, [face-to-face care] could be compared with a [digital therapeutic]. However, I do think it’s something that would be helpful when there’s no supports available and things like that.

Young person 06; aged 16 years

However, health professionals indicated that body language cannot be monitored via digital tools as easily as in person. In addition, further clarification on issues raised by young people cannot be sought in instances of self-guided digital therapeutics. Health professionals advised that it would be harder to assess risk or provide personalized therapeutic intervention via digital therapeutics:

I don’t think digital technologies can replace face-to-face counselling sessions.... It’s difficult to interpret body language. It’s difficult to fully interpret the nonverbal communication that people are letting off. You run the risk of making some errors in your judgment about someone’s safety.

Health professional 07; physician

Young people explained that interpersonal connection made them feel safe and were concerned that this could not be provided by a digital therapeutic:

However, it wouldn’t be the same, I think, as talking to another person. And because even though it can be scary, social interaction is important when you’re in really dark moments because it just proves to you that you’re not alone.... Whereas an app, I don’t think would be able to give off that same vibe or safety.

Young person 12; aged 17 years

In addition, some young people and health professionals indicated that engaging with a digital therapeutic was less confronting than difficult and sensitive conversations face-to-face with strangers and, therefore, would have a place in assessing young people to decide on a care pathway:

I mean, in saying that some young people prefer face-to-face, so it’s when you have that young person presenting to the emergency department or wherever, if they’re unwilling to engage with you, there’s that option there for them to engage with [a digital therapeutic].... But definitely offer the face-to-face as well. I don’t think we can ever shy away from face-to-face because there’s such great value in that.

Health professional 01; mental health clinician

##### Digital Therapeutics Might Be Inappropriate in a Crisis

Both young people and health professionals noted that, when individuals experience a heightened level of distress during a suicide crisis, they are unlikely to respond to a digital therapeutic. Limited evidence for the effectiveness of digital therapeutics in comparison with face-to-face services was identified as a barrier to the provision of a digital therapeutic in a hospital. However, young people and health professionals noted that a digital therapeutic could be more appropriate once the height of the crisis had passed—such as after discharge:

It’s really hard because I feel like if you get into ED [emergency department] you’re at that level and you’re about to explode. They’re not going to be able to read that stuff, they’re going to be like, “Piss off, just let me see someone.”

Health professional 04; nurse

I feel like when I was in my mindset, what I was in hospital, I just didn’t want anything. I just wanted to be dead. I just didn’t want to do anything. I wasn’t listening. I was refusing to do anything really, because I was just in that mindset of nothing’s going to change, and stuff.

Young person 16; aged 16 years

##### Digital Therapeutics Are Always Accessible

Young people observed that digital devices are an easier way to administer and access information and support compared with face-to-face treatment:

I feel like it’d be a really good way to have such a big pack of resources in one app, that you can access, and have a lot of information, and stuff, that your hands on your phone. Because a lot of people these days are on their phone quite a lot. So, if you’ve got that right on your hands, it’s really handy.

Young person 16; aged 16 years

Young people highlighted the benefit of accessing digital therapeutics when they felt most ready and competent to engage with the intervention. This was particularly important for young people who might wane in and out of high distress during the hospital visit:

I don’t know, you could zone out. But like if you have something right there it’s not going anywhere.... You can zone out as much as you want it’s still going to be there.

Young person 02; aged 16 years

Young people also highlighted the all-hour accessibility of digital therapeutics. This was particularly helpful as clinicians might not work at a hospital and hospital wait times might be long:

I’ve had no one to connect with kind of thing, and covering those resources while I’m [at the hospital] that would be extremely helpful.

Young person 06; aged 16 years

Young people also saw value in digital therapeutics once out of the hospital to receive care in the community. This is because they noted that digital therapeutics are more accessible than traditional mental health services, which often have long waitlists and high costs:

It’s a lot more convenient. I mean, you don’t have to go out of your way, drive, pay for petrol, sit in a waiting room...it’s there. You can use it when you have to. It’s not scheduled for this time every week. You can use it throughout the week, which would definitely help a lot more people than if you just had an appointment every couple weeks.

Young person 12; aged 17 years

#### Theme 2: Barriers to Investing Time, Energy, and Work on the Intervention (Cognitive Participation)

This theme clarified the barriers that hindered engagement with or delivery of the intervention. These included limited access to information and communications technology as well as time and human resource constraints.

##### Limited Access to Information and Communications Technology

Health professionals and young people identified limited access to a device or charger as barriers to the use of a digital therapeutic. Young people also mentioned insufficient Wi-Fi in hospitals, particularly in emergency departments. The cost of purchasing smartphone apps could also hinder access:

Because I mean the other thing is if they just ask them to download the apps, that might not even be possible because sometimes the person might not have a phone with them, might not have internet, might run out of credit or something. So, they might not be even able to download an app.

Health professional 03; social worker

Young people and health professionals indicated that one solution was the provision of a hospital-owned tablet to access the digital therapeutic:

So it might be possible that there will be some waiting area and then there’s some sort of iPads available and then the triage nurse can introduce [it]...And then they can of browse it and then they can practice it.

Health professional 03; mental health clinician

However, some young people reported that they would feel uncomfortable using a hospital device to work through a digital therapeutic for a sensitive topic such as suicide, and they had privacy concerns. As such, young people recommended that there be an option to use either their own device or the hospital tablet. Furthermore, young people wanted the option to download the digital therapeutic to their own device if using a hospital tablet to continue to use the digital therapeutic after discharge:

I think, yeah, with that, if they gave me a device, then in my mind I would be like, “Is everything that I’m doing on this being monitored?”

Young person 14; aged 22 years

##### Time and Human Resource Constraints

Health professionals commented that insufficient staffing availability significantly hinder the adoption of new practices. High patient-to-staff ratios reduced time to provide care and health professional capacity to engage with additional practices to existing care:

So obviously the key thing is communication and involving the staff in that process. So the worst thing is to go, “Here’s a new app, you’ve got to use it, here’s...” And the staff just kind of go, “I’m already busy enough and it’s hard enough to do my job, you give me something else to do.”

Health professional 08; peer support worker

#### Theme 3: Compatibility Considerations for Integration Into Existing Workplace Practices or Infrastructure (Collective Action)

This theme clarified the considerations required when delivering a digital therapeutic in a hospital. They included when a digital therapeutic should be offered in the hospital journey, how hospital staff do or do not support digital therapeutic use, alternative technology options to access the digital therapeutic, and the importance of framing the digital therapeutic as optional.

##### Timing

Both health professionals and young people preferred a digital therapeutic to be offered in the waiting room or emergency department, with little support for use in a hospital inpatient setting. This was largely due to the long waiting times that participants often experienced at this junction of the visit:

Waiting room, pass the time. Yeah, because, I mean, realistically the wait times in emergency departments are sort of blowing out. The quicker people could relieve that distress, then why wouldn’t we do it?

Health professional 02; nurse

I just think when you’re waiting. You can be waiting to be triaged for a long time...once you’ve been triaged...they could say, “Here’s this thing, have a look at it while you wait.” That’s the time that needs to be filled. And that’s the time where you’re lying there, thinking, like, “How did I get myself here?” Blaming yourself, feeling really guilty, feeling awful about all of it.

Young person 11; aged 23 years

Young people also supported the provision of a digital therapeutic on, or just before, discharge. However, some noted that this would decrease engagement with a digital therapeutic, particularly after the long wait to access care before discharge:

I think it would be helpful, but it needs to be done quite tactfully, I think. Because I think if you’re in quite a vulnerable space and someone just says, “Oh, download this app.” That might not be received very well. I think especially if you do have quite invalidating experiences and then you get sent home with an app saying, “Sorry, we can’t help you, but...”

Young person 14; aged 22 years

##### Supported Use of Digital Therapeutics

For young people, being provided a digital therapeutic with some assistance from staff members to initially show them how to use the intervention and then just to occasionally check in with the young person was the most favorable of all delivery formats:

I think, like, for the first like 10-15 minutes of being introduced to the app, having someone who will walk you through it and help you identify the particular skills you want to be focusing in on in the app I think that would be helpful...

Young person 05; aged 22 years

I think I’d like to do it on my own, but I’d like them to check in, stuff like that.

Young person 08; aged 16 years

I think personally, I’d like hospital staff to come over and just kind of give me the rundown of how it works. Even just to comfort you, like you’re okay, you’re safe now. And probably do it by myself from then on.

Young person 12; aged 17 years

Health professionals also noted that some degree of staff assistance would be beneficial. This was largely because young people in a suicide crisis often need interpersonal connection. Staff also recognized that young people might be more inclined to use a digital therapeutic if they have an opportunity to understand its value and ask relevant questions:

I think that people need connections with people...I want to sit down, and I just want to show [young people] what we’ve got that I can give [them] right now and walk them through it and show them how valuable it [is].

Health professional 12; peer support worker

Health professionals also saw digital therapeutics as an opportunity to provide information to young people about their patient journey, which they hypothesized would create feelings of safety:

So being able to just maybe pass over, or put someone in a room with an iPad, or a computer system, or an app on their phone, and just saying it in a way as, “The mental health team here would like you to have a check out this app before you go into the assessment today.” It’s going to give them a bit of a head start on what’s going on for you, and that really puts them in a safe space because then they don’t have to go into the conversation any further with them.

Health professional 06; mental health clinician

##### Framed as Optional

Young people reported that they were more likely to engage with a digital therapeutic if it was offered as optional rather than mandatory. This originated from a desire for privacy and the experience of involuntary hospital admission with their autonomy restricted:

Maybe just leave it on the bed or on the table, or whatever. It’s like left on, and just have it there and then it’ll be like, “Just use it. If you want to. You don’t have to be. If not we’ll be back in half an hour to see if you’re using it or not.” I feel like, if they just trusted me with it straight up. I’d be like, “Okay? Well, maybe I’ll just have a look at it. I’m with the curious because I’m bored,” you know.

Young person 07; aged 18 years

#### Theme 4: What Is Needed for Staff to Integrate Into Practice (Collective Action)

This theme clarified what health professionals considered important for a digital therapeutic to be integrated into routine practice in hospitals. These included increasing staff support for the digital therapeutic, conducting ongoing training, engaging local opinion leaders, and informing staff of changes.

##### Increasing Staff Support

Health professionals recognized that increasing staff support for the digital therapeutic would facilitate engagement and improve uptake. Health professionals identified three ways to achieve this: (1) involving staff in decisions to ensure that the intervention fits their workplace, (2) promoting the benefits to staff, and (3) showing the evidence for why the intervention is beneficial to patients. This suggests that health professionals are most motivated to use a new practice if it is perceived to enhance their capacity to support their patients within their time and resource constraints:

Well, the only onerous part is time. The time invested in encouraging someone to have an engagement with a digital technology needs to have a benefit for the patient and for the flow in the department to be worth the time expended to do it. If you can reduce someone’s hospital stay length by a period of time and make them safe for discharge, then that’s going to be.... I mean, really in ED we are so understaffed, and we are so busy, and we are so short of beds that most decisions come down to what’s going to allow us to make safe decisions most quickly. Anything that assists with that will be beneficial.

Health professional 07; physician

##### Conducting Ongoing Training

Health professionals noted that frequent and ongoing training is already integrated into the hospital system to support ongoing skill development and build awareness of new and ongoing hospital processes. Health professionals identified that staff training was essential to support the implementation of digital therapeutics in hospitals to give staff an opportunity to learn, engage, and ask questions to ensure that they are comfortable offering the digital therapeutic:

Just making sure good education about its provided and I think that’d be one of the main things and I think with that they’d all get around it for sure.

Health professional 04; nurse

There were differing opinions about whether training should be conducted face-to-face, with the opportunity to practice using the digital therapeutic themselves, or whether didactic information about the digital therapeutic was sufficient. There was no consensus on whether training should be mandatory or optional; however, health professionals highlighted that it was essential that they be notified of changes to the digital therapeutic in some way to ensure that they could continue to understand how young people would be using digital therapeutics:

Maybe the chatting to staff about it and maybe running training days where everyone gets to have a go at using it and having it be more of a hands-on thing rather than a concept, rather than coming and being like, “I’ve got this concept. This is what it is.”

Health professional 05; nurse

I think just information is sufficient. I think if you try and do mandatory training that’s going to impede your implementation and also people do mandatory training on so many things that it’s probably going to reduce the likelihood that it would be used. Most departments have a morning brief and it’s better just to say, “Hey, we’ve got this new thing,” as part of the morning brief where we tell people that tell staff about new things that are happening in the department or other concerns and things.

Health professional 07; physician

##### Local Opinion Leaders

According to the health professionals, practice change is best facilitated when senior staff encourage implementation of an intervention and staff engagement via a “top-down” model of innovation diffusion. This is largely due to their hierarchical influence and their ability to oversee the implementation to ensure that all staff are informed and supported:

...what gets things onboard is having senior high regard staff because it’s so hierarchical, you need the doctors to be onboard with it because if you are having your senior doctors telling you that this is beneficial, we need to do this, you’re going to have everybody else follow through with those things, and people that are pushing it and using it.

Health professional 06; mental health clinician

##### Informing Staff of Changes

Health professionals identified a need to inform staff of implementation changes. This can facilitate team awareness of the intervention, an understanding of its benefits, and engagement:

We’re all on the same page. Everyone understood what was happening. People had chances to ask questions and try it out and practice using it and yeah, that was really good.

Health professional 05; nurse

In addition, health professionals identified the need for continued reminders of how and when to use the digital therapeutic and to keep staff aware of updates and changes to the digital therapeutic and its implementation:

Once it’s implemented, then you can do app updates and you can send the emails or you can do, “Hey, we’re doing a big group refresher course on just the updates” and you could get everyone there for if there’s five new pages or whatever. I think that it’s worthwhile taking that time...

Health professional 12; peer support worker

## Discussion

### Principal Findings

This study explored the perspectives of health professionals and young people to understand whether and how to implement digital therapeutics for suicide prevention in hospitals. There was strong agreement between these 2 stakeholder groups regarding how they collectively “made sense” of digital innovations and what they identified as potential barriers to, and drivers of, implementation. Although digital innovations were seen as beneficial insomuch as they may improve access to care and reduce the risk of young people falling through the gaps, there was consensus that they should complement but not replace face-to-face care. The entry and discharge points of the patient journey were seen as the most appropriate opportunities for young people to be offered these innovations, but their benefits may be best realized when used as a “community-based” aftercare support after discharge. A key factor in adoption or normalization was having a clear “top-down” leadership and governance model driven by senior staff and supported by training provisions and use reminders. Building the “digital health literacy” of staff was also emphasized by young people, who indicated a strong preference for staff initially working through digital innovations with them as part of sense making and engagement. Our findings highlight a number of implementation challenges and illustrate the importance of involving end users and providers as health innovations are being conceptualized to identify and design solutions that could practicably work to support normalization.

Emergency departments are a common entry point into health care for young people in suicidal distress but are increasingly recognized as inappropriate and potentially harmful settings for individuals in acute states of psychological distress to receive care [6,7]. These settings are often crowded, noisy, and likely to lack private and safe spaces to discuss sensitive issues [31]. Emergency departments are also not structurally designed for empathetic and humanistic care. Performance expectations are that >80% of patients will be admitted or discharged within 4 hours of presenting to an emergency department [32] such that “care” in these settings can realistically only be short and sharp. Despite our findings showing that young people and staff perceive there to be benefits of integrating digital innovations into existing models of care, the sociopolitical environments and constraints in which hospitals operate mean that implementation is likely to be both technically and humanistically challenging. Only the beginning (at presentation) and the end (at discharge) of the patient journey were considered to be appropriate touchpoints for delivery, with in-person (human) care being recognized as critical to recovery for young people admitted to hospital. These findings are consistent with those of previous studies that have examined potential uses of digital interventions for suicide prevention [13,33,34]. However, although digital innovations could be offered at the point of presentation to support young people in emotionally regulating during potentially extensive wait times to assessment [35,36], and despite evidence that these can be effective tools for reducing symptoms of suicidal ideation [37], depression, and anxiety [38], it may also be inappropriate to do so. At the point of presentation, young people often experience reduced cognitive capacity [39], including because of intoxication, and would likely find it difficult to engage with a self-directed digital therapeutic innovation. Providing digital innovations as a model of aftercare might be the most appropriate pathway to normalization as cost, technical, and cognitive barriers are less prominent at this stage compared with at earlier stages of the hospital journey.

Young people preferred digital innovations that were more aligned with self-guided or “direct-to-consumer”–type models that would allow them to independently explore content at their own pace. This finding is consistent with those of previous studies in which personal autonomy and flexibility were found to be important considerations for young people in how they engage with digital health interventions [40,41]. Although staff time constraints were identified as a barrier to implementation in this study and elsewhere [42], both stakeholder groups suggested that having a specifically trained staff member to provide brief education on how to access and use these tools and to answer questions would be critical to successful implementation. As Graham et al [15] argue, providing “education-focused” implementation supports is an important ethical consideration for digital innovation adoption as these are emerging (new) practices that young people may not be familiar with and, therefore, may not benefit properly from without guidance. However, there was a consensus that staff should not be required to have a prolonged, ongoing role in delivery. Limiting the role and time required from staff to deliver digital therapeutics is necessary as workforce and resource capacity issues in Australian hospitals are widely recognized as key barriers to innovation adoption [19]. Dedicating resources toward digital therapeutics is likely to be a low priority right now, particularly while the evidence base for the effectiveness of these innovations in suicide prevention is still emerging [12,40]. It will be important to accelerate research efforts that not only focus on effectiveness but also demonstrate that digital innovations offer practical value with respect to streamlining mental health care pathways, improving patient flow, and enhancing patient care experiences. Such evidence is required to garner support at all levels of health care provision and create buy-in to digital mental health care practices. The role of lived-experience peer workforces in building capacity to deliver these innovations in hospital settings, including at discharge, should be considered as a potentially important implementation strategy.

The limited access to information and communications technology within emergency departments and variable Wi-Fi quality were consistently identified as barriers to implementation. If digital innovations are to have a place in the emergency department, hospitals may need to provide tablets to patients, with the option of installing digital innovations on personal devices. Such structural considerations may matter less if digital innovations are limited to being offered only as an aftercare option as patients would then naturally access these innovations via their own devices.

Having top-down leadership to drive the implementation of new models of care that incorporate digital innovations was seen as important by hospital staff. Effective communication of any practice changes from senior leaders to all staff was seen as imperative for implementation, as was ensuring that brief and ongoing training was provided as part of a standard rollout model. Although implementation research specific to suicide prevention remains scarce, these findings are consistent with one previous study that examined the implementation of a clinical suicide prevention intervention in an Australian health setting—which also identified training, leadership, communication, and support as key determinants that could help or hinder the sustainability of a new therapeutic approach [41]. As the broader digital mental health implementation literature suggests, implementation supports would need to be purposefully tailored to the unmet needs and contexts of individual hospitals, with consideration given to the volume and complexity of young people presenting in a suicidal crisis, hospital workflows and processes to identify consumers [42], resourcing and infrastructure, and the optimal touchpoints for delivery (eg, aftercare only vs presentation or admission) [15]. However, although implementation variance will be inevitable, investigating the training needs and literacy gaps of staff, and understanding where commonalities exist across hospitals, may be helpful for identifying opportunities to standardize and digitize core components of training and supporting resources. This approach could help minimize the burden on hospitals to develop lengthy bespoke training programs and enable the scalability of key resources.

### Strengths and Limitations

Our study has several strengths. First, we used an implementation science framework (NPT) to guide the interviews, chosen specifically as it is designed to shape the translation of innovations in health care systems. This ensured that participant responses clarified the processes and conditions necessary to understand how a digital therapeutic could become part of routine practice. Second, data were collected from both consumers and providers of digital therapeutics to obtain a thorough understanding of the appropriateness and feasibility of the intervention. Third, data were collected from young people and health professionals from a variety of Australian states to capture a breadth of experiences with receiving and delivering care in hospitals. Fourth, health professionals were selected from a variety of roles (eg, nurses, mental health clinicians, peer workers, and physicians) to obtain a more comprehensive understanding of the perceived usability of digital therapeutics for different hospital staff members.

There were also some limitations to our study. First, most of the health professionals (7/12, 58%) had no experience delivering digital therapeutics, meaning that their recommendations for how to optimize implementation were hypothetical. Second, to protect young people’s anonymity, they were not asked to identify the hospital they attended, limiting our ability to draw comparisons between the hospital settings that youth and hospital participants were referring to. The data could have been affected by self-selection bias in that individuals who were already interested in digital therapeutics in a hospital setting opted to participate, and therefore, our data may not have captured the full extent of barriers to implementation. Finally, young people (potential beneficiaries) were not involved in the development of the interview guide, and as such, we may have missed asking questions about implementation that were not part of the NPT framework. Although it is undoubtedly valuable to use established implementation frameworks to guide data collection, future studies should work collaboratively with end users and implementation workforces to refine interview guides and enhance the depth of perspective captured.

### Conclusions

This study highlights the importance of early-stage scoping of the appropriateness of new health practice innovations before large-scale implementation, contributing much needed evidence to the intersecting fields of suicide prevention and implementation science. Although there was clear agreement that digital therapeutics may offer a unique and beneficial way to support young people who experience suicide crises in hospitals, several important considerations emerged that would need to be addressed before these tools are embedded into existing hospital practices. Our findings advance current understandings of how health innovations could enhance care in “real-world” contexts and provide directions for the types of implementation strategies that could help support adoption in real-world health settings.

## Abbreviations

COREQ: Consolidated Criteria for Reporting Qualitative Research
NPT: Normalization Process Theory

## Data Availability

The data set generated and analyzed during this study is not publicly available because of privacy considerations but is available from the corresponding author upon reasonable request.

## References

[1] Hawton K, Saunders KE, O'Connor RC (2012). Self-harm and suicide in adolescents. Lancet.

[2] Perera J, Wand T, Bein KJ, Chalkley D, Ivers R, Steinbeck KS, Shields R, Dinh MM (2018). Presentations to NSW emergency departments with self-harm, suicidal ideation, or intentional poisoning, 2010-2014. Med J Aust.

[3] John J, Synn EP, Winata T, Eapen V, Lin PI (2023). Increased ambulance attendances related to suicide and self-injury in response to the pandemic in Australia. Aust N Z J Psychiatry.

[4] Sara G, Wu J, Uesi J, Jong N, Perkes I, Knight K, O'Leary F, Trudgett C, Bowden M (2023). Growth in emergency department self-harm or suicidal ideation presentations in young people: comparing trends before and since the COVID-19 first wave in New South Wales, Australia. Aust N Z J Psychiatry.

[5] Hill NT, Witt K, Rajaram G, McGorry PD, Robinson J (2021). Suicide by young Australians, 2006-2015: a cross-sectional analysis of national coronial data. Med J Aust.

[6] Byrne SJ, Bellairs-Walsh I, Rice SM, Bendall S, Lamblin M, Boubis E, McGregor B, O'Keefe M, Robinson J (2021). A qualitative account of young people's experiences seeking care from emergency departments for self-harm. Int J Environ Res Public Health.

[7] Freeman J, Strauss P, Hamilton S, Pugh C, Browne K, Caren S, Harris C, Millett L, Smith W, Lin A (2022). They told me "this isn't a hotel": young people's experiences and perceptions of care when presenting to the emergency department with suicide-related behaviour. Int J Environ Res Public Health.

[8] Cully G, Leahy D, Shiely F, Arensman E (2022). Patients' experiences of engagement with healthcare services following a high-risk self-harm presentation to a hospital emergency department: a mixed methods study. Arch Suicide Res.

[9] Geulayov G, Casey D, Bale L, Brand F, Clements C, Farooq B, Kapur N, Ness J, Waters K, Tsiachristas A, Hawton K (2019). Suicide following presentation to hospital for non-fatal self-harm in the Multicentre Study of Self-harm: a long-term follow-up study. Lancet Psychiatry.

[10] Braciszewski JM (2021). Digital technology for suicide prevention. Adv Psychiatry Behav Health.

[11] Büscher R, Torok M, Terhorst Y, Sander L (2020). Internet-based cognitive behavioral therapy to reduce suicidal ideation: a systematic review and meta-analysis. JAMA Netw Open.

[12] Torok M, Han J, Baker S, Werner-Seidler A, Wong I, Larsen ME, Christensen H (2020). Suicide prevention using self-guided digital interventions: a systematic review and meta-analysis of randomised controlled trials. Lancet Digit Health.

[13] Melia R, Francis K, Hickey E, Bogue J, Duggan J, O'Sullivan M, Young K (2020). Mobile health technology interventions for suicide prevention: systematic review. JMIR Mhealth Uhealth.

[14] Brådvik L (2018). Suicide risk and mental disorders. Int J Environ Res Public Health.

[15] Graham AK, Lattie EG, Powell BJ, Lyon AR, Smith JD, Schueller SM, Stadnick NA, Brown CH, Mohr DC (2020). Implementation strategies for digital mental health interventions in health care settings. Am Psychol.

[16] Triplett NS, Woodard GS, Johnson C, Nguyen JK, AlRasheed R, Song F, Stoddard S, Mugisha JC, Sievert K, Dorsey S (2022). Stakeholder engagement to inform evidence-based treatment implementation for children's mental health: a scoping review. Implement Sci Commun.

[17] Geerligs L, Rankin NM, Shepherd HL, Butow P (2018). Hospital-based interventions: a systematic review of staff-reported barriers and facilitators to implementation processes. Implement Sci.

[18] Kirchner JE, Parker LE, Bonner LM, Fickel JJ, Yano EM, Ritchie MJ (2012). Roles of managers, frontline staff and local champions, in implementing quality improvement: stakeholders' perspectives. J Eval Clin Pract.

[19] Pomare C, Churruca K, Long JC, Ellis LA, Braithwaite J (2019). Organisational change in hospitals: a qualitative case-study of staff perspectives. BMC Health Serv Res.

[20] May C, Finch T (2009). Implementing, embedding, and integrating practices: an outline of normalization process theory. Sociology.

[21] May CR, Cummings A, Girling M, Bracher M, Mair FS, May CM, Murray E, Myall M, Rapley T, Finch T (2018). Using normalization process theory in feasibility studies and process evaluations of complex healthcare interventions: a systematic review. Implement Sci.

[22] Richards JE, Simon GE, Boggs JM, Beidas R, Yarborough BJ, Coleman KJ, Sterling SA, Beck A, Flores JP, Bruschke C, Grumet JG, Stewart CC, Schoenbaum M, Westphal J, Ahmedani BK (2021). An implementation evaluation of "Zero Suicide" using normalization process theory to support high-quality care for patients at risk of suicide. Implement Res Pract.

[23] Owens C, Charles N (2016). Implementation of a text-messaging intervention for adolescents who self-harm (TeenTEXT): a feasibility study using normalisation process theory. Child Adolesc Psychiatry Ment Health.

[24] Blattert L, Armbruster C, Buehler E, Heiberger A, Augstein P, Kaufmann S, Reime B, Rural Suicide Prevention Study Group (2022). Health needs for suicide prevention and acceptance of e-mental health interventions in adolescents and young adults: qualitative study. JMIR Ment Health.

[25] Tong A, Sainsbury P, Craig J (2007). Consolidated criteria for reporting qualitative research (COREQ): a 32-item checklist for interviews and focus groups. Int J Qual Health Care.

[26] Boudreaux ED, Jaques ML, Brady KM, Matson A, Allen MH (2015). The patient safety screener: validation of a brief suicide risk screener for emergency department settings. Arch Suicide Res.

[27] Griffith R (2016). What is Gillick competence?. Hum Vaccin Immunother.

[28] Huddlestone L, Turner J, Eborall H, Hudson N, Davies M, Martin G (2020). Application of normalisation process theory in understanding implementation processes in primary care settings in the UK: a systematic review. BMC Fam Pract.

[29] Gale NK, Heath G, Cameron E, Rashid S, Redwood S (2013). Using the framework method for the analysis of qualitative data in multi-disciplinary health research. BMC Med Res Methodol.

[30] Ritchie J, Lewis J, Nicholls CM, Ormston R (2013). Qualitative Research Practice: A Guide for Social Science Students and Researchers. 2nd edition.

[31] Asarnow JR, Babeva K, Horstmann E (2017). The emergency department: challenges and opportunities for suicide prevention. Child Adolesc Psychiatr Clin N Am.

[32] Sullivan C, Staib A, Khanna S, Good NM, Boyle J, Cattell R, Heiniger L, Griffin BR, Bell AJ, Lind J, Scott IA (2016). The National Emergency Access Target (NEAT) and the 4-hour rule: time to review the target. Med J Aust.

[33] Sarubbi S, Rogante E, Erbuto D, Cifrodelli M, Sarli G, Polidori L, Lester D, Berardelli I, Pompili M (2022). The effectiveness of mobile apps for monitoring and management of suicide crisis: a systematic review of the literature. J Clin Med.

[34] Witt K, Spittal MJ, Carter G, Pirkis J, Hetrick S, Currier D, Robinson J, Milner A (2017). Effectiveness of online and mobile telephone applications ('apps') for the self-management of suicidal ideation and self-harm: a systematic review and meta-analysis. BMC Psychiatry.

[35] Quinlivan L, Gorman L, Littlewood DL, Monaghan E, Barlow SJ, Campbell S, Webb RT, Kapur N (2022). 'Wasn't offered one, too poorly to ask for one' - reasons why some patients do not receive a psychosocial assessment following self-harm: qualitative patient and carer survey. Aust N Z J Psychiatry.

[36] Rheinberger D, Macdonald D, McGillivray L, Maple M, Torok M, Nicolopoulos A, Shand F (2021). "A sustained, productive, constructive relationship with someone who can help"-a qualitative exploration of the experiences of help seekers and support persons using the emergency department during a suicide crisis. Int J Environ Res Public Health.

[37] Torok M, Han J, McGillivray L, Wong Q, Werner-Seidler A, O'Dea B, Calear A, Christensen H (2022). The effect of a therapeutic smartphone application on suicidal ideation in young adults: findings from a randomized controlled trial in Australia. PLoS Med.

[38] Seegan PL, Miller MJ, Heliste JL, Fathi L, McGuire JF (2023). Efficacy of stand-alone digital mental health applications for anxiety and depression: a meta-analysis of randomized controlled trials. J Psychiatr Res.

[39] Ellis TE, Rutherford B (2008). Cognition and suicide: two decades of progress. Int J Cogn Ther.

[40] Barasa EW, Molyneux S, English M, Cleary S (2015). Setting healthcare priorities in hospitals: a review of empirical studies. Health Policy Plan.

[41] Zbukvic I, Rheinberger D, Rosebrock H, Lim J, McGillivray L, Mok K, Stamate E, McGill K, Shand F, Moullin JC (2022). Developing a tailored implementation action plan for a suicide prevention clinical intervention in an Australian mental health service: a qualitative study using the EPIS framework. Implement Res Pract.

[42] Mogk JM, Matson TE, Caldeiro RM, Garza Mcwethy AM, Beatty T, Sevey BC, Hsu CW, Glass JE (2023). Implementation and workflow strategies for integrating digital therapeutics for alcohol use disorders into primary care: a qualitative study. Addict Sci Clin Pract.

